# The Effect of Renal Artery Stent Implantation on Clinical Outcomes in Patients with Early-Stage (Non-Atrophic Kidney) and Clinically Overt Severe Atherosclerotic Renal Artery Stenosis (ARAS-TR)

**DOI:** 10.3390/jcm15103825

**Published:** 2026-05-15

**Authors:** Mehmet Kış, Fatih Levent, Mehmet Altunova, Sadık Volkan Emren, Mustafa Doğduş, Beytullah Çakal, Oktay Şenöz, Tuncay Güzel, Çisem Oktay, Ömer Faruk Kahraman, Sezgin Atmaca, Yunus Emre Erata, Tumarzat Ulanbekova, Mehmet Birhan Yılmaz

**Affiliations:** 1 Department of Cardiology, Dokuz Eylul University Faculty of Medicine, 35340 Izmir, Türkiye; cisemoktay25@gmail.com (Ç.O.); birhan.yilmaz@deu.edu.tr (M.B.Y.); 2 Department of Cardiology, University of Health Sciences Bursa Yuksek Ihtisas Training and Research Hospital, 16330 Bursa, Türkiye; fatihlevent85@hotmail.com (F.L.); dromerfaruk95@gmail.com (Ö.F.K.); 3 Department of Cardiology, Mehmet Akif Ersoy Thoracic and Cardiovascular Surgery Training and Research Hospital, 34303 Istanbul, Türkiye; dr.mehmetaltunova@gmail.com (M.A.); sezgin_3570@hotmail.com (S.A.); yemre.er@gmail.com (Y.E.E.); 4 Department of Cardiology, Izmir Kâtip Çelebi University Faculty of Medicine, 35620 Izmir, Türkiye; vemren@hotmail.com (S.V.E.); www.tumarzat@gmail.com (T.U.); 5 Department of Cardiology, Izmir University of Economics Faculty of Medicine, 35330 Izmir, Türkiye; mdogdus@hotmail.com; 6 Department of Cardiology, Medipol University of Health Sciences, 34810 Istanbul, Türkiye; bcakal@medipol.edu.tr; 7 Department of Cardiology, University of Bakırçay, 35665 Izmir, Türkiye; oktay.senoz@bakircay.edu.tr; 8 Department of Cardiology, Diyarbakır Provincial Health Directorate Gazi Yaşargil Education and Research Hospital, 21070 Diyarbakır, Türkiye; drtuncayguzel@gmail.com

**Keywords:** renal artery stenosis, atherosclerosis, hypertension, stent implantation, clinical outcome

## Abstract

**Objective:** Atherosclerotic renal artery stenosis (ARAS) is increasingly prevalent among aging populations and in patients with diabetes, hyperlipidemia, aortoiliac obstructive disease, coronary artery disease, and/or hypertension. Patients with severe ARAS are at a substantially elevated risk of cardiovascular disease, recurrent congestive heart failure, stroke, ischemic nephropathy, and chronic kidney disease. Therefore, the ARAS-TR study aims to evaluate the effect of renal artery stenting on the clinical outcomes in patients with severe ARAS and renovascular hypertension. **Materials:** This study was conducted as a multicenter, prospective study between July 2024 and September 2025. It encompassed 278 patients with angiographically confirmed severe ARAS who underwent renal artery stent implantation. Patients were subsequently monitored for 6 months. A paired-samples *t*-test was used to compare continuous variables pre- and post-intervention, while categorical variables were analyzed using the Pearson chi-square test and Fisher’s exact test. **Results:** The mean age of the patients was 63.6 [±13.4] years, and the male gender ratio was 52.5%. After renal artery stenting, systolic and diastolic blood pressures decreased significantly at the 6-month follow-up compared with the pre-procedure levels (SBP 166.99 [21.24] vs. 135.40 [15.69], *p* < 0.001; DBP 96.28 [13.03] vs. 80.39 [11.03], *p* < 0.001, respectively). GFR (61.23 [28.33] vs. 63.35 [26.36], *p* = 0.029) and creatinine (1.40 [0.93] vs. 1.29 [0.66], *p* = 0.004) levels improved compared to baseline. The mean number of antihypertensive drugs required for patients to remain normotensive decreased significantly (3.19 [1.04] vs. 2.48 [1.13], *p* < 0.001) during the follow-up period. **Conclusions:** Percutaneous renal artery intervention appears to be a promising and safe strategy for carefully selected high-risk patients presenting with severe ARAS, renovascular hypertension, and non-atrophic kidneys. In this specific clinical context, restoring renal artery patency through percutaneous stenting was associated with improved renal function and observed reduction in the burden of antihypertensive drugs required to sustain normotension.

## 1. Introduction

The predominant cause of renal artery stenosis (RAS) remains atherosclerosis. Atherosclerotic renal artery stenosis (ARAS) is increasingly observed in elderly populations and among individuals with diabetes, hyperlipidemia, aortoiliac obstructive disease, coronary artery disease (CAD), or hypertension (HT) [1,2]. Patients presenting with severe ARAS are often associated with a substantially elevated risk of cardiovascular disease, recurrent congestive heart failure, stroke, ischemic nephropathy, and chronic kidney disease (CKD) [1,3]. ARAS could be considered in patients exhibiting treatment-resistant hypertension, recurrent pulmonary edema, and a progressive decline in renal function [1,4]. ARAS most commonly occurs in the ostium and proximal segment of the renal artery [2]. Although renal Doppler ultrasound (RDUS), computed tomography (CT), and magnetic resonance imaging (MRI) are frequently employed diagnostic modalities, none are considered definitive for establishing the diagnosis of ARAS. When diagnostic tests indicate suspicion of ARAS, selective renal angiography, regarded as the gold standard diagnostic procedure, is often warranted for definitive evaluation [1,2,5].

Renovascular hypertension (RVH) is a condition in which renal artery occlusion or significant renal artery stenosis reduces renal perfusion pressure to a level that activates the renin–angiotensin–aldosterone system, thereby raising blood pressure [1]. RVH is the leading cause of secondary hypertension. Patients with severe RVH generally have a poor response to antihypertensive treatment [6]. Currently, three therapeutic options are available for RVH patients with ARAS: antihypertensive therapy, surgical revascularization, and percutaneous transluminal angioplasty/stent implantation [1,2,6]. Severe ARAS can result in resistant HT, acute pulmonary edema, and CKD and/or renal atrophy due to ischemic nephropathy. Patients presenting with these clinical features have been suggested to potentially achieve a more favorable blood pressure response following renal artery stent implantation [3].

Several randomized clinical trials (RCTs) investigating the addition of renal artery stenting to medical therapy in patients with ARAS did not demonstrate additional clinical benefit [7,8,9]. However, these landmark trials have been subject to discussion regarding certain methodological considerations. A notable consideration is the potential underrepresentation of patients with high-risk clinical features, such as severe renovascular disease. In many of these studies, inclusion criteria led to the enrollment of cohorts with mild RAS, stable hypertension, or advanced chronic kidney disease with marked renal atrophy—these included patient groups that may inherently be less responsive to revascularization. Consequently, patients presenting with clinical features suggestive of functionally significant RAS, such as acute pulmonary edema, resistant hypertension, or rapid decline in renal function following the administration of an angiotensin-converting enzyme inhibitor or angiotensin receptor blocker, were largely excluded [7,8,9]. This suggests that the clinical utility of renal artery stenting may be more evident in a carefully selected, high-risk patient population, as explored in the present study.

In patients with severe ARAS, regular follow-up examinations are crucial after optimal medical treatment and/or renal artery revascularization. Follow-up should include laboratory tests to assess renal function, analysis of office and outpatient blood pressure recordings (ambulatory or home blood pressure monitoring as per the ESC Guidelines for arterial hypertension), and renal artery Doppler ultrasonography [2].

This study aims to evaluate the effect of renal artery stenting on clinical outcomes in patients with severe atherosclerotic renal artery stenosis and renovascular hypertension.

## 2. Materials and Methods

### 2.1. Study Design and Patient Populations

This study was designed as a multicenter and prospective observational study conducted at eight centers specializing in peripheral interventional procedures. Initially, 281 consecutive patients with angiographically confirmed severe ARAS who underwent percutaneous renal artery stent implantation between July 2024 and September 2025 were enrolled. Of these, 3 patients were excluded from the final analysis due to missing 6-month follow-up data. Consequently, the study was completed with a final cohort of 278 patients. Both procedural details and clinical outcomes were collected prospectively. All included patients completed a standardized 6-month clinical follow-up prior to data analysis in March 2026.

### 2.2. Inclusion and Exclusion Criteria

Inclusion criteria for the study: History of acute pulmonary edema or acute decompensation of heart failure (HF) with severe ARAS, or progressive chronic renal failure in the presence of severe ARAS, or acute renal failure due to acute renal artery occlusion or severe ARAS, or intolerance to ACE inhibitors (ACEi) or angiotensin receptor blockers (ARBs) with severe ARAS, or resistant hypertension with angiographically documented > 70% stenosis of renal artery. Patients presenting with acute coronary syndrome, severe liver failure, active malignancy, and acute ischemic stroke, patients presenting with cardiogenic shock, patients on dialysis, and patients with findings of renal atrophy or significant cortical thinning in their imaging studies (CT, MRI, or Ultrasound) were excluded from the study.

In this study, ‘early-stage’ was defined based on the morphological preservation of the renal parenchyma rather than the duration of clinical symptoms. As specified in the exclusion criteria, patients with findings of renal atrophy or marked cortical thinning on imaging (CT or Ultrasound) were excluded to ensure the focus remained on salvageable renal tissue. Consequently, the study population consisted of patients with severe arterial stenosis but preserved (non-atrophic) kidneys, representing a critical clinical window for intervention where functional recovery is more likely.

### 2.3. Procedure

Selective renal artery angiography is the gold- standard method for diagnosing RAS and allows for additional hemodynamic measurements. In all patients, selective renal angiography was the main diagnostic procedure. Detection of >70% stenosis in at least one renal artery was considered severe stenosis [2]. Since ARAS usually originates from large aorto-ostial plaques, balloon angioplasty alone is generally ineffective due to rebound associated with these large plaques; renal artery stent implantation is the preferred primary revascularization strategy [10].

Weight-adjusted unfractionated heparin was administered to all patients to achieve an intraprocedural activated clotting time (ACT) of 250–300 s. Stent implantation was performed in all patients. Technical success was defined as a residual diameter stenosis of <20% with preserved distal TIMI 3 flow. Post-procedural care included standard dual antiplatelet therapy and monitoring of puncture site integrity and renal function. All patients were followed up for 6 months.

This study was conducted in accordance with the principles of the Declaration of Helsinki and has been approved by the Ethics Committee (Decision number: 1643, Date: 3 July 2024).

### 2.4. Definitions of Clinical Parameters

The patients’ age, gender, body mass index, blood pressure values, smoking history, comorbidities, and treatments received, as well as echocardiographic parameters and laboratory results, were recorded in the relevant sections of the case report form. Diagnosis of left ventricular diastolic dysfunction (LVDD) was made by expert, independent echocardiographers at each center, as per guidelines [1,11]. Left ventricular hypertrophy (LVH) was defined according to recent guidelines [1].

All patients were maintained on maximally tolerated medical therapy prior to the intervention. To address potential confounding in clinical outcomes, post-procedural medication adjustments followed a standardized, protocol-driven approach rather than being at the sole discretion of the physician, whereby dose reductions were initiated only upon the achievement of office blood pressure targets (<140/90 mmHg) or the occurrence of symptomatic hypotension. In patients classified as NYHA Class III–IV, renal artery stenting will be performed only after clinical stabilization of acute heart failure symptoms is achieved. These individuals are managed with optimized intravenous or oral medical therapy until they become hemodynamically stable and are capable of tolerating the percutaneous intervention. Furthermore, to ensure consistency in reporting, an ‘ex-smoker’ was defined as any individual who had successfully refrained from smoking for at least one year prior to the baseline evaluation. Images related to renal angiography and stent(s) implantation were reviewed by two independent, experienced interventional cardiologists.

### 2.5. Statistical Analysis

All statistical analyses were performed using the SPSS for Windows version 15.0 software (SPSS Inc., Chicago, IL, USA). The Kolmogorov–Smirnov test was used to check for normality of distribution for continuous variables. Continuous variables were presented in mean ± standard deviation (SD), while categorical variables were presented in number and frequency. A paired-sample *t*-test was used to compare continuous variables before and after the renal stent. Categorical variables were compared using the Pearson chi-square and Fisher’s exact test. Pre- and post-follow-up comparisons of antihypertensive medication status were performed using the McNemar test. A *p*-value of <0.05 was considered statistically significant.

## 3. Results

### 3.1. Clinical Characteristic Features

All patients (n = 278) had >70% stenosis in at least one renal artery. Mean age was 63.6 [±13.4] years, and the male gender ratio was 52.5%. In the study, 104 (37.4) patients had stage II–III HT, and 36% had CKD. There was non-obstructive accompanying coronary artery disease (CAD) in 134 (48.2%) patients and obstructive CAD in 90 (32.4%) patients. The current smoking rate was 32.4%. Demographic and comorbid characteristics of the study population are summarized in Table 1.

### 3.2. Imaging and Stent Procedure Findings

Electrocardiography revealed left ventricular hypertrophy according to the Sokolow-Lyon criteria in 82 (29.5%) patients. Echocardiographic findings showed left ventricular diastolic dysfunction in 123 (44.2%) patients. Accessory renal arteries were detected in 8.3% of the patients who underwent the procedure. The mean diameter of the implanted stents was 5.82 [±0.93] mm for the right renal artery and 5.81 [±0.91] mm for the left renal artery. In all centers, only peripheral balloon-expandable renal stents were used for the stenting procedure. The imaging and angioplasty procedure findings of the patients are given in Table 2.

### 3.3. Evaluation of the Effect of Renal Stent Procedure on Laboratory and Imaging Findings

After renal stenting, systolic and diastolic pressures decreased significantly at the 6-month follow-up compared to the pre-procedure level (SBP 167 [±21.93] vs. 135 [±15.69] *p* < 0.001, DBP 96 [±13.03] vs. 80 [±11.03] *p* < 0.001, respectively). The echocardiographically assessed E/e’ value decreased significantly (11.14 [±3.21] vs. 10.21 [±3.26], *p* < 0.001). Estimated GFR (61.23 [±28.33] vs. 63.35 [±26.36], *p* = 0. 029) and creatinine (1.40 [±0.93] vs. 1.29 [±0.66], *p* = 0.004) values were improved compared to the baseline levels. Data regarding the evaluation of clinical outcomes of renal stent implantations are summarized in Table 3.

### 3.4. Antihypertensive Medications Used by Patients Before the Procedure and During the Follow-Up Period

The average number of blood pressure-lowering drugs used by patients decreased significantly during the follow-up period (3.19 [±1.04] vs. 2.48 [±1.13], *p* < 0.001). According to the McNemar test performed between paired categorical variables, significant differences were detected across specific drug classes. Regarding these specific pharmacological classes, the administration of ACEi and ARBs significantly declined from baseline to follow-up (39.2% to 33.8%, *p* = 0.002, and 39.9% to 34.2%, *p* < 0.001, respectively). A substantial reduction was observed in the administration of beta-blocker (79.5% to 60.1%, *p* < 0.001) and CCB usage (65.8% to 47.8%, *p* < 0.001). Notably, alpha-blocker prescription rates were markedly diminished (20.9% to 11.5%, *p* < 0.001), and diuretic treatment strategies exhibited a sharp decrease from 61.2% to 20.9% at the 6-month visit (*p* < 0.001). In contrast, the use of ARNI remained constant (0.7% at both time points, *p* = 1), and no statistically significant change was reached for MRA (14.7% to 10.1%, *p* = 0.096). The antihypertensive medications used by patients before the procedure and during the follow-up period are listed in Table 4.

### 3.5. Procedure-Related Complications and Adverse Events Occurring During Follow-Up

Renal artery perforation was reported to occur in 3 (1.1%) patients during the renal stenting procedure, and significant residual stenosis remained in 2 (0.7%) patients. Stent thrombosis occurred in 3 (1.1%) patients during the post-procedure follow-up period. The femoral artery was used as the access site for the procedure in all patients, and major bleeding complications occurred at the access site in 10 (3.6%) patients.

All-cause mortality rate during the follow-up was 5.04%. Cardiovascular hospitalizations were observed in 21 (7.55%) patients, and HF hospitalizations were observed in 23 (8.27%) patients. During the post-procedure follow-up, 13 (4.68%) patients were admitted to the emergency department with hypertensive crisis, and 12 (4.32%) patients had pulmonary edema at admission. Procedure-related complications and adverse events occurring during follow-up are summarized in Table 5.

### 3.6. Figures

**Figure 1:**
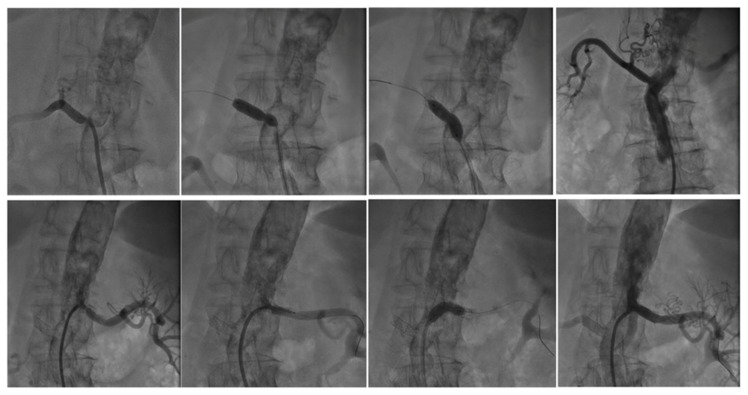
A 74-year-old female patient. CAD (history of multiple PCI procedures), HT, and porcelain aorta present. Patient exhibited shortness of breath and resistant hypertension. Bilateral selective renal angiography revealed 75% ostial stenosis in the right renal artery and 80% ostial stenosis in the left renal artery. A 6.0 × 18 mm renal balloon-expandable stent was implanted in the right renal artery ostial stenosis. A 6.0 × 18 mm renal balloon-expandable stent was implanted in the left renal artery ostial stenosis. Since complete patency was not achieved in the left renal artery ostial stenosis, the stent balloon was withdrawn, and post-dilatation was performed under high pressure. Patience was achieved.

**Figure 2:**
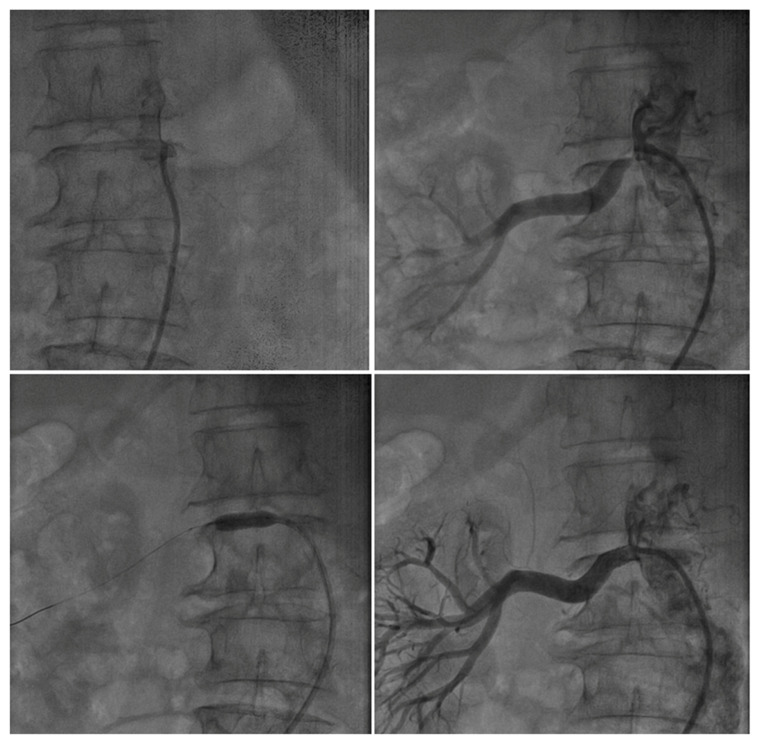
A 73-year-old female patient. Comorbidities included HT, DM, CAD (history of multiple PCI procedures), and CKD. Recent history of emergency department presentation due to HT and HF-related AC edema. Creatinine levels have elevated from 1.3 to 1.6 over the past month. Bilateral selective renal angiography revealed 95–99% stenosis in the right renal ostium and total occlusion of the left renal artery. CT angiography showed the left kidney was atrophic, with no perfusion observed; no blood flow was detected; therefore, no intervention was performed for the total occlusion of the left renal artery. Pre-dilatation of the right renal artery stenosis was performed with a 4.0 × 12 mm balloon, followed by implantation of a 6.0 × 14 mm balloon-expandable renal stent at 14 atm pressure. Patience was achieved.

Two examples in the ARAS-TR study, a challenging intervention for bilateral severe renal artery stenosis in a patient with resistant hypertension and porcelain aorta (Figure 1) and unilateral renal artery intervention in a patient with severe ARAS presenting to the emergency department with pulmonary edema (Figure 2), are presented below.

## 4. Discussion

The ARAS-TR study provides clinical insights into the importance of timely intervention in a high-risk cohort, observing significant decrease in blood pressure and a noteworthy decrease in the average number of antihypertensive medications during the 6-month follow-up. Furthermore, enhancements were noted in renal functional parameters, suggesting a potential role for revascularization in specifically targeted populations.

ARAS is not a well-defined entity. Its incidence is estimated to range from 30% in patients with coronary artery disease to 50% in the elderly or those with extensive atherosclerotic vascular disease. ARAS is a progressive disease that can occur alone or in combination with hypertension and ischemic heart disease [6,12,13,14]. Guidelines recommend lifestyle modification and medical treatment as the first option. However, percutaneous transluminal renal angioplasty/stent implantation and surgical treatment options are also available for selected patients [1,2,15]. The goal of revascularization is to control blood pressure, potentially prevent/slow the progression of renal failure, and favorably influence improve cardiovascular outcomes. Atherosclerotic lesions respond poorly to balloon angioplasty alone due to significant residual stenosis and restenosis. In this situation, stent implantation is recommended for patients with resistant hypertension, worsening renal function, and/or treatment-resistant heart failure, as well as recurrent pulmonary edema, as it may improve both immediate and long-term outcomes [6,7,13,14].

Several large studies have sought to test the superiority of standard medical treatment plus renal artery angioplasty in lowering blood pressure or preventing adverse renal and cardiovascular outcomes compared with medical treatment alone. Stent placement in patients with atherosclerotic renal artery stenosis and impaired renal function (STAR) (140 patients with RAS ≥ 50%) [7] and Revascularization versus Medical Therapy for Renal-Artery Stenosis (ASTRAL) (806 patients) [9] showed no significant difference in blood pressure levels nor progression of renal disease in patients treated with percutaneous transluminal renal angioplasty (PTRA) and standard medication compared to patients treated with medication alone. Finally, in the multicenter Stenting and Medical Therapy for Atherosclerotic Renal-Artery Stenosis (CORAL) study, which included 947 patients with a median follow-up of 43 months, there was no additional benefit over medical treatment alone in terms of blood pressure control, renal function, or adverse cardiovascular outcomes [8]. These studies have been subject to ongoing debate regarding their methodological frameworks. These studies had non-standardized inclusion criteria, which led to the inclusion of a large number of patients with advanced CKD who had mild/asymptomatic RAS, mild hypertension, or atrophic kidneys groups that may inherently be less responsive to RAS revascularization. Additionally, the RCTs mentioned above regarding renal stenting almost entirely excluded patients with a clinical presentation suggestive of functionally significant RAS, such as acute pulmonary edema, refractory hypertension, or rapid loss of renal function following ACEi or ARB use. In addition, revascularization is not recommended in cases of mild-to-moderate stenosis and/or a totally occluded renal artery [13].

In a recent non-randomized study of 102 patients with renal artery stenosis and severe atherosclerotic RAS who presented high-risk clinical features, 96 patients with 3-month follow-up data experienced a mean reduction in 24 h ambulatory systolic blood pressure of 19.6 mm Hg (95% CI, 15.4–23.8; *p* < 0.001). The daily dose of antihypertensive medication decreased by 52% (95% CI, 41–62%; *p* < 0.001), and the estimated glomerular filtration rate increased by 7.8 mL/min per 1.73 m^2^ (95% CI, 4.5–11.1; *p* < 0.001) [6]. A non-randomized study involving 208 patients examined the safety and longevity of renal stenting after suboptimal or failed renal artery angioplasty in individuals with suspected renovascular hypertension. Results showed that systolic/diastolic blood pressure decreased from 168 [±25]/82 [±13] mm Hg at baseline to 149 [±24]/77 [±12] mm Hg at 9 months (*p* < 0.001) and remained at 149 [±25]/77 [±12] mm Hg at 24 months (*p* < 0.001). The mean serum creatinine level stayed stable at both 9 and 24 months compared to initial values. In hypertensive patients with aorto-ostial atherosclerotic renal artery stenosis, balloon-expandable stents have been shown to provide a safe and permanent revascularization strategy with a beneficial effect on hypertension [16]. In a single-center retrospective study of 78 ARAS patients who underwent renal artery stenting, a significant decrease in serum creatinine (8.50 μmol/L) was observed during follow-up (*p* < 0.05). Among the four subgroups, patients with mild renal impairment showed an increase in eGFR (10.68 mL) during follow-up (*p* < 0.01). In the remaining patients with other stages of renal function, no significant change in eGFR was observed before and after renal stenting [17]. In the ARAS-TR multicenter study, eGFR (61.23 [±28.33] vs. 63.35 [±26.36], *p* = 0.029) and creatinine (1.40 [±0.93] vs. 1.29 [±0.66], *p* = 0.004) improved from baseline. Furthermore, in the ARAS-TR study, there was a more substantial decrease in systolic and diastolic blood pressures at the six-month follow-up compared to the pre-procedure levels (SBP 166.57 [±21.93] vs. 135.40 [±15.69], *p* < 0.001; DBP 96.28 [±13.03] vs. 80.39 [±11.03], *p* < 0.001), consistent with and extending the findings of the non-randomized studies previously mentioned.

Complications of percutaneous renal artery angioplasty and/or stent implantation are rare but have been reported [6,14,18]. The complication rates in the ARAS-TR study are low, suggesting that renal artery stenting can be considered a safe procedure. Of note, contrast nephropathy occurs in less than 5% of patients undergoing percutaneous renal artery stenting [18,19]. The low incidence of post-procedure contrast nephropathy (10 [3.59%]) in the ARAS-TR study may be attributed to routine pre- and post-procedure saline hydration and to the enrollment of patients with relatively better kidney function.

High event rates are documented among patients undergoing stenting for RAS [3,16,20]. A retrospective analysis utilizing data from a single-center, single-operator renal stent study, known as SOCRATES, encompassed 748 consecutive symptomatic patients with newly diagnosed ARAS. During hospitalization, mortality rates at 30 days and 6 months were 0.5%, 2.0%, and 6.3%, respectively [20]. In the multicenter ARAS-TR study, the all-cause mortality rate during the follow-up period was reported as 5.04%, with no mortality occurring during the in-hospital period. Cardiovascular hospital admissions occurred in 21 patients (7.55%), and HF hospitalization occurred in 23 patients (8.27%) during the follow-up period. In the follow-up period, the high mortality rate reflects the clinical characteristics of our real-world cohort, which included patients with severe ARAS and life-threatening presentations such as acute decompensated heart failure, acute pulmonary edema, and/or refractory hypertension, who are frequently excluded from major randomized controlled trials.

While landmark randomized trials and systematic reviews—including CORAL [8], ASTRAL [9], and the Cochrane review by Jenks et al. [21]—have highlighted the challenges in demonstrating a universal clinical advantage for revascularization in unselected ARAS populations, our findings provide supplemental insights into specific high-risk groups. Although the absence of a control group warrants a cautious interpretation regarding causality, the favorable outcomes observed in the ARAS-TR study are likely attributable to patients with high-risk, severe renal artery stenosis, and non-atrophic kidneys. This particular cohort was often underrepresented or excluded in earlier large-scale trials, suggesting that meticulous patient selection remains a critical factor in achieving strong clinical responses to percutaneous renal intervention.

Consequently, the ARAS-TR study is primarily limited by its observational, single-arm design, which lacks a randomized comparison with optimal medical therapy alone. This inherent methodological constraint warrants a cautious interpretation regarding definitive causality, as our findings primarily reflect the clinical experiences within this specific cohort. It should be emphasized that the primary focus of the ARAS-TR study was to evaluate the procedural safety and clinical efficacy of renal artery stenting within a high-risk cohort. While the current data demonstrate observed significant variations in hemodynamic parameters and stabilization of renal function through paired analyses, multivariable regression was not performed to preclude the risk of statistical overfitting given the focused sample size. Consequently, a more granular investigation—specifically a comparative analysis of clinical responders versus non-responders and the identification of independent predictors of major adverse cardiovascular events—was deemed beyond the scope of this index report. To maintain methodological rigor, these comprehensive evaluations are currently being conducted as dedicated sub-studies within the ARAS-TR project. These forthcoming analyses are expected to provide deeper insights into the specific clinical phenotypes that may derive the most profound benefit from revascularization, thereby further refining the criteria for stringent patient selection in the management of severe ARAS.

The 6-month follow-up period is relatively brief, which inherently limits our ability to evaluate the long-term durability of the clinical benefits and late-term complications. However, it should be emphasized that this duration was strategically selected to prioritize the assessment of acute and mid-term clinical stabilization in high-risk patients. Additionally, the inclusion of a highly selected high-risk population introduces potential selection bias, which may limit the generalizability of our findings to the broader ARAS population. However, this focus provides critical ‘real-world’ evidence for a symptomatic segment that has historically been underrepresented or excluded in large-scale randomized trials. The observed ‘modest’ renal benefits are consistent with the natural history of ARAS, where the primary therapeutic objective in advanced cases is to stabilize renal function and the prevention of further ischemic decline rather than the complete restoration of glomerular filtration rate. Blood pressure was monitored in the office; however, the lack of ambulatory blood pressure monitoring is one of the study’s limitations. Although the operators were experienced with the peripheral interventions, the differences between operators may have affected the rates of procedure-related complications and outcomes due to the multicenter nature of the study. Ultimately, ARAS-TR provides real-world evidence suggesting the potential clinical utility of revascularization when targeted at patients who historically represent the most symptomatic and high-risk segment of the ARAS spectrum, reflecting a possible synergistic benefit between procedural success and intensive medical management.

## 5. Conclusions

Percutaneous renal artery intervention appears to be a promising and safe strategy for carefully selected high-risk patients presenting with severe ARAS, renovascular hypertension, and non-atrophic kidneys. In this specific clinical context, restoring renal artery patency through percutaneous stenting was associated with improved renal function and observed reduction in the burden of antihypertensive drugs required to sustain normotension. Nevertheless, the absence of a comparison arm necessitates further randomized trials to confirm these potential benefits specifically in this high-risk population.

## Figures and Tables

**Table 1:** Demographic and comorbid characteristics of patients.

Demographic and Comorbid Parameters	n (%)
Age, years (mean standard ± deviation)	63.6 [±13.4]
Male gender	146 (52.5)
NYHA	
I	104 (37.4)
II	123 (44.2)
III	44 (15.8)
IV	7 (2.5)
Chest pain	124 (44.6)
Palpitation	48 (17.3)
Dyspnea	132 (47.5)
Headache	118 (42.4)
Syncope	4 (1.4)
Flushing	14 (5.0)
Abdominal murmur	60 (21.6)
Hypertension	
Grade I	174 (62.6)
Grade II	59 (21.2)
Grade III	45 (16.2)
Diabetes Mellitus	135 (48.5)
CAD	
None	54 (19.4)
Non- obstructive	134 (48.2)
Obstructive	90 (32.4)
CAD Treatments	
Medical	71 (25.5)
PCI	117 (42.1)
CABG	40 (14.4)
Atrial fibrillation	28 (10.1)
CVD	26 (9.4)
Chronic renal failure	100 (36.0)
COPD	41 (14.7)
Anemia	38 (13.7)
Thyroid disease	23 (8.3)
Lower extremity PAD	48 (17.3)
Carotid stenosis	
None	232 (83.5)
<50%	34 (12.2)
>50%	1 (0.4)
Unknown	11 (4.0)
Smoking	
No	140 (50.4)
Yes	90 (32.4)
Ex-smoker	48 (17.3)
Alcohol	33 (11.9)

CAD: coronary artery disease, CABG: Coronary Artery Bypass Graft, COPD: Chronic Obstructive Pulmonary Disease, CVD: cerebrovascular disease, NYHA: New York Heart Association, PAD: Peripheral artery disease, PCI: Percutaneous Coronary Intervention.

**Table 2:** Imaging and stenting procedure findings of the study population.

ECG and Echocardiography Findings	n (%)
LVH	82 (29.5)
LVDD	123 (44.2)
**Renal Angiography/Stenting Procedure Findings**	**n (%)**
Accessory renal artery	23 (8.3)
Renal artery aneurysm	2 (0.7)
Renal artery ectasia	1 (0.4)
Right renal artery stent diameter, mm, (mean + Std)	5.82 [0.93]
Right renal artery stent length, mm, (mean + Std)	16.66 [3.47]
Left renal artery stent diameter, mm, (mean + Std)	5.81 [0.91]
Left renal artery stent length, mm, (mean + Std)	16.82 [3.85]

**Table 3:** Evolution of Hemodynamic, Echocardiographic, and Renal Functional Parameters from Baseline to 6-Month Follow-up.

	Baseline	6 Month	*p* Value
SBP	166.99 [21.24]	135.40 [15.69]	**<0.001**
DBP	96.28 [13.03]	80.39 [11.03]	**<0.001**
Pulse	74.82 [11.41]	74.68 [10.69]	0.833
LVEF	55.56 [9.38]	55.82 [9.32]	0.470
Ascending Aorta	35.72 [4.16]	35.62 [4.04]	0.518
LVEDD	47.85 [5.02]	47.54 [4.70]	0.142
LVESD	29.06 [6.21]	31.30 [6.06]	**<0.001**
IVs	12.94 [1.91]	12.65 [1.92]	**0.002**
Pw	11.98 [1.71]	11.85 [1.72]	0.139
LA	39.80 [5.39]	39.94 [5.38]	0.492
E/e’	11.14 [3.21]	10.21 [3.26]	**<0.001**
Urea	28.83 [17.08]	26.98 [14.21]	**0.030**
Creatinine	1.40 [0.93]	1.29 [0.66]	**0.004**
Uric acid	6.19 [1.83]	6.18 [1.70]	0.906
GFR	61.23 [28.33]	63.35 [26.36]	**0.029**

SBP: Systolic blood pressure, DBP: Diastolic blood pressure, GFR: Glomerular filtration rate, IVs: Interventricular septum, LA: Left atrium, LVEDD: Left ventricle end-diastolic diameter, LVEF: Left ventricle ejection fraction, LVESD: Left ventricle end-systolic diameter, Pw: Posterior wall.

**Table 4:** Comparison of Antihypertensive Medication Profiles and Drug Burden at Baseline and 6-Month Follow-up.

	Baseline	6 Month	*p* Value
ACE-I	108 (39.2)	94 (33.8)	**0.002**
ARB	111 (39.9)	95 (34.2)	**<0.001**
ARNI	2 (0.7)	2 (0.7)	1
BB	221 (79.5)	167 (60.1)	**<0.001**
MRA	41 (14.7)	28 (10.1)	0.096
CCB	183 (65.8)	133 (47.8)	**<0.001**
Alpha-blocker	58 (20.9)	32 (11.5)	**<0.001**
Diuretic	170 (61.2)	116 (41.7)	**<0.001**
Furosemide	44 (15.8)	35 (12.6)	
Torsemide	11 (4.0)	10 (3.6)	
Indapamide	23 (8.3)	13 (4.7)	
Thiazide	92 (33.1)	58 (20.9)	
Total antihypertensive drug	3.19 [1.04]	2.48 [1.13]	**<0.001**

ACE-I: Angiotensin-converting-enzyme inhibitor, ARB: Angiotensin II receptor blockers, ARNI: Angiotensin Receptor-Neprilysin Inhibitor, BB: Beta blocker, CCB: Calcium channel blockers, MRA: Mineralocorticoid receptor antagonists.

**Table 5:** Complications and Major Adverse Clinical Events During the Follow-up Period.

Complications	n (%)
Stent dislodgement	0 (0)
Stent thrombosis	3 (1.1)
Significant residual stenosis	2 (0.7)
Access site hematoma	18 (6.5)
Pseudoaneurysm	4 (1.4)
Renal artery perforation	3 (1.1)
Major access site bleeding	10 (3.6)
Contrast nephropathy	10 (3.6)
**Adverse Event at Follow-Up**	**n (%)**
Hospitalization due to CAD	21 (7.55)
Hospitalization due to HF	23 (8.27)
Cerebrovascular disease	9 (3.24)
Hypertensive Crisis	13 (4.68)
Acute pulmonary edema	12 (4.32)
All-cause mortality	14 (5.04)

## Data Availability

The datasets presented in this study are not publicly available due to multi-center confidentiality and ethical restrictions. The data presented in this study are available on request from the corresponding author.

## Abbreviations

The following abbreviations are used in this manuscript:

ARAS	Atherosclerotic renal artery stenosis
CABG	Coronary Artery Bypass Graft
CAD	Coronary artery disease
GFR	Glomerular filtration rate
CKD	Chronic kidney disease
CVD	Cerebrovascular disease
NYHA	New York Heart Association
PAD	Peripheral artery disease
PCI	Percutaneous Coronary Intervention
